# Tumor Microenvironment and Microvascular Density in Follicular Lymphoma

**DOI:** 10.3390/jcm11051257

**Published:** 2022-02-25

**Authors:** Roberto Tamma, Giuseppe Ingravallo, Tiziana Annese, Francesco Gaudio, Tommasina Perrone, Pellegrino Musto, Giorgina Specchia, Domenico Ribatti

**Affiliations:** 1 Department of Basic Medical Sciences, Neurosciences, and Sensory Organs, University of Bari Medical School, Piazza G. Cesare 11, 70124 Bari, Italy; tiziana.annese@uniba.it; 2 Department of Emergency and Transplantation, Pathology Section, University of Bari Medical School, 70124 Bari, Italy; giuseppe.ingravallo@uniba.it; 3 Department of Emergency and Transplantation, Hematology Section, University of Bari Medical School, 70124 Bari, Italy; francesco.gaudio@uniba.it (F.G.); tommasina.perrone@policlinico.ba.it (T.P.); pellegrino.musto@uniba.it (P.M.); giorgina.specchia@uniba.it (G.S.)

**Keywords:** angiogenesis, follicular lymphoma, NHL, tumor microenvironment

## Abstract

Follicular lymphoma (FL) is a slowly progressive disease and constitutes the second most common non-Hodgkin lymphoma. Biological factors, such as the tumor microenvironment and the host response, are determinants in the outcome of FL but the experimental data about microenvironment and tumor cells in FL are variable and contradictory. In this morphometric study, we analyzed by immunohistochemistry the cellular components of the tumor microenvironment and correlated these data with the microvascular vascular density in three different grades of FL lymph node biopsies, comparing the results to healthy lymph node controls. The results indicated a significant increase in the number of CD68^+^ and CD163^+^ macrophages in all three analyzed FL grades. Tryptase^+^ mast cells resulted in an increase only in grade 1. PDL-1^+^ cells, CD4- and CD8-lymphocytes number results were reduced in FL samples. The higher number of CD34^+^ microvessels in the FL grades 1 and 2 of samples positively correlated with CD68^+^ and CD163^+^ cells, underlining the important angiogenic potential of this subset of macrophages.

## 1. Introduction

Follicular lymphoma (FL) is a slowly progressive disease belonging to B-cell non-Hodgkin lymphomas (B-NHLs), constituting the second most common NHL. The median age is around 60 with a male to female ratio of 1:1.7. Usual sites of disease development principally include the lymph nodes, but may involve the spleen, bone marrow, peripheral blood, and gastrointestinal tract [1,2]. Its indolent progression leads to delayed diagnosis and consequently, a high percentage of patients present an advanced FL at the initial diagnosis. FL affected patients show a variable outcome in absence of treatment, including spontaneous remissions reported in about 15% of the patients [3].

FL results from the clonal expansion of germinal center (GC) B cells, even if malignant transformation is initiated during early B-cell development in the bone marrow, by aberrant repair failure of V(D)J recombination. The resulting t(14;18) translocation promotes high expression of the anti-apoptotic protein BCL2 [4], providing a survival advantage to B cells during GC reaction, wherein BCL2 is normally actively repressed by the GC-specific transcriptional machinery [5,6].

According to the World Health Organization (WHO) classification, FL includes grade 1–3 (low grade to high grade) depending on the percentage of centroblasts: grade 1 (follicular small cleaved): 0–5 centroblasts/high-power field (HPF), grade 2 (follicular mixed): 6–15 centroblasts/HPF, and grade 3 (follicular large cell): > 15 centroblasts/HPF (grade 3A—centrocytes present, grade 3B—solid sheets of centroblasts). It is important to clinically differentiate grades 1/2 vs. grade 3 [2,7]. In half of FL patients, a histological transformation to aggressive lymphoma such as diffuse large B cell lymphoma (DLBCL) occurs, inducing a worse outcome [8]. The FL treatments are variable and include watchful waiting and the anti-CD20 antibody as monotherapy, a combination of chemotherapy, radiotherapy, and radioimmunotherapy, or chemotherapy at a high dosage followed by hematopoietic stem cell transplantation. Promising results have emerged with the anti-CD20 antibody and chemotherapy association [9].

Although it is well documented that genetic changes are involved in FL initiation and progression, the interaction between the immune microenvironment and FL tumor cells is acquiring increasing importance [10,11,12]. In FL patients, neoplastic follicles maintain an architecture similar to the normal lymph nodes in which B cells remain dependent on cellular and molecular events that contribute to the normal GC and include stromal cells, T-follicular helper cells (Tfh), T-follicular regulatory cells (TFRs), and follicular dendritic cells (FDCs), normally found in the lymphoid follicles [13].

The prognosis of FL remains heterogeneous, and this negatively contributes to the programming of an effective clinical design. Many prognostic factors have been considered allowing the creation of the FL International Prognostic Index (FLIPI) to develop a more accurate prognostic index. FLIPIs are based on clinical factors but they ignore biological factors, such as the tumor microenvironment (TME) and the host response [14,15]. It is well known that all the steps following tumorigenesis including the growth, progression, and metastatic process, are strongly influenced by the tumor microenvironment [16,17]. TME is a complex environment surrounding the cancer cells that includes cellular and extracellular components and a vascular network [18] which are also involved in the determination of therapeutic efficacy [19].

Scientific works highlight the importance of the TME composition in determining the pathogenesis of lymphoma and the complex interplay between the different cells of TME. Tumor associated macrophages (TAMs) promote immunosuppression inhibiting the recruitment and activity of T cells [20]. Moreover, TAMs influence tumor progression by regulating angiogenesis [21]. Mast cells are also involved in tumor progression [22,23] through pro-tumoral, stimulating angiogenesis and anti-tumoral responses and recruiting other immune cells [24]. CD8^+^ T cells have been reported in FL TME with an inconstant number and are mainly located in the perifollicular regions of the lymph node [25,26]. Furthermore, CD8^+^ T cell counts are lower than CD4^+^ T cells. FL tumor cells modulate gene expression of CD8^+^ and CD4^+^ cells [27], and the survival of FL tumor cells is dependent on surrounding CD4^+^ T cells [28,29]. Moreover, CD4^+^ T cells’ heterogeneity in FL and their distribution play an independent role in prognosis and permitting integration into prognostic scores to improve risk stratification [30]. Finally, CD4^+^ and CD8^+^ cells mediate the anti-angiogenic effect of IL-12 [31] and express IFNγ which inhibits the proliferation of endothelial cells and induces the expression of CXCL9/10/11 in TAMs with an angiostatic effect [32,33].

TME is enabling the discovery of targeted therapies and providing data to improve the prediction of tumor progression. For example, PD-1/PD-L1 controls excessive immunity of cytotoxic T cells leading to the failure of T cell immunity. Tumor cells express PD-L1 [34], and as a consequence of PD-1/PD-L1 pathway inhibition, T cells become active and exert more pronounced antitumor effects by rescuing exhausted T cells [35,36]. In B-cell lymphoma, the PD-1/PD-L1 blockade therapy has shown positive effects promoting the formation of “hot” immune-inflamed TME [37,38]. Combination treatment, for example, with chimeric antigen receptor T cells (CAR-T) and nivolumab, or PD-1 inhibition with concomitant radiotherapy can then be used [39].

Pembrolizumab is the only FDA-approved agent for use with R/R B-NHL (i.e., PMBL) patients [40]. Nivolumab was evaluated in patients with relapsed and refractory B-NHL and a phase I study of 54 patients with NHL, including 10 FL, 11 DLBCL, 10 other B cell lymphomas, 13 peripheral T cell lymphoma (PTCL), and 5 other T cell lymphomas [41]. The highest ORR was observed among FL patients, followed by DLBCL. In the phase I study of ipilimumab in patients with R/R B-NHL, 18 patients were enrolled [42]. Two patients had clinical responses; the ORR was low, only 11%. One with DLBCL achieved a durable CR lasting >31 months, and another with FL had a PR lasting 19 months. In a phase I study, patients with R/R FL received nivolumab (1 or 3 mg/kg, every 2 weeks) [43].

The data in the literature about the interactions between the microenvironment and tumor cells in FL are variable and contradictory although it is thought that TME in FL acts in two directions, one supporting tumor growth and survival and the other suppressing the anti-tumoral immune response. Immunohistochemical analysis on FL tissue samples is bringing out important information on FL TME cell populations as well as their spatial distribution in the tissue.

In this work, we analyzed, by an immunohistochemical approach, the lymph node biopsies derived from FL patients at grades 1,2, and 3A, at first diagnosis and focused our attention on the quantification of tumor microenvironmental cells, including macrophages, mast cells, CD4^+^ and CD8^+^ lymphocytes, and PD-L1 positive cells and the microvessel density in the different groups of patients comparing them with normal lymph nodes.

## 2. Materials and Methods

### 2.1. Patients

This retrospective study included bioptic specimens derived from 60 lymph nodes of FL patients of 1, 2, and 3A grade at first diagnosis (Table 1) and 20 healthy lymph nodes used as control. The 60 patients and the 20 controls have been subdivided into FL1, FL2, FL3 groups according to the FL grade and CTRL group. The samples were collected from the archive of the Section of Pathology of the University of Bari School of Medicine between 2019 and 2021. All procedures were in accordance with the ethical standards of the responsible committee on human experimentation (institutional and national) and with the Helsinki Declaration of 1964 and later versions; signed informed consent from individual patients were obtained to conduct the study.

### 2.2. CD4, CD8, CD68, CD163, Tryptase, CD34, and PDL-1 Immunohistochemistry

Serial histological sections of 4 mm thickness, collected on poly-L-lysine-coated slides (Sigma Chemical, St. Louis, MO, USA), were deparaffinized. The sections were rehydrated in a xylene-graded alcohol scale and then rinsed for 10 min in 0.1 M PBS. Sections were pretreated with sodium citrate (pH 6.1) solution (DAKO, Glostrup, Denmark) for antigen retrieval for 30 min at 98 °C and then incubated with mouse monoclonal anti-CD4 (DAKO, Glostrup, Denmark), mouse monoclonal anti-CD8 (DAKO, Glostrup, Denmark), mouse monoclonal anti-CD68 (DAKO, Glostrup, Denmark), mouse monoclonal anti-CD163 (DAKO, Glostrup, Denmark), mouse monoclonal anti-Tryptase (DAKO, Glostrup, Denmark), mouse monoclonal anti-CD34 (DAKO, Glostrup, Denmark), and mouse monoclonal anti-pd-l1 (DAKO, Glostrup, Denmark) diluted 1:50, 1:50 1:100, 1:100, 1:100, 1:100, and 1:50, respectively. Thereafter, the sections were counterstained with Mayer hematoxylin and mounted in a synthetic medium. Specific pre-immune serum (DAKO), replacing the primary antibodies, served as a negative control. Ten sections from each experimental group were scanned using the whole-slide morphometric analysis scanning platform Aperio Scanscope CS (Leica Biosystems, Nussloch, Germany). All the slides were scanned at the maximum available magnification (40×) and stored as digital high-resolution images on the workstation associated with the instrument. Digital slides were inspected with Aperio ImageScope v.11 software (Leica Biosystems, Nussloch, Germany) at 20× magnification, and 10 fields with an equal area were selected for the analysis at 40× magnification. The protein expression was assessed with the Positive Pixel Count algorithm embedded in the Aperio ImageScope software and reported as positivity percentage, defined as the number of positively stained pixels on the total pixels in the image.

### 2.3. Statistical Analysis

Data related to the three experimental groups, FL1, FL2, and FL3, are reported as means ± SE. Newman–Keuls multiple comparisons post-test was used to compare all treatment groups after one-way ANOVA. The Graph Pad Prism 5.0 statistical package (GraphPad Software, San Diego, CA, USA) was used for analyses and the limit for statistical significance was set at *p* < 0.05. Correlation analysis was performed with the Spearman nonparametric correlation test.

## 3. Results

### 3.1. CD68, CD163, and Tryptase Immunohistochemistry

**Figure 1:**
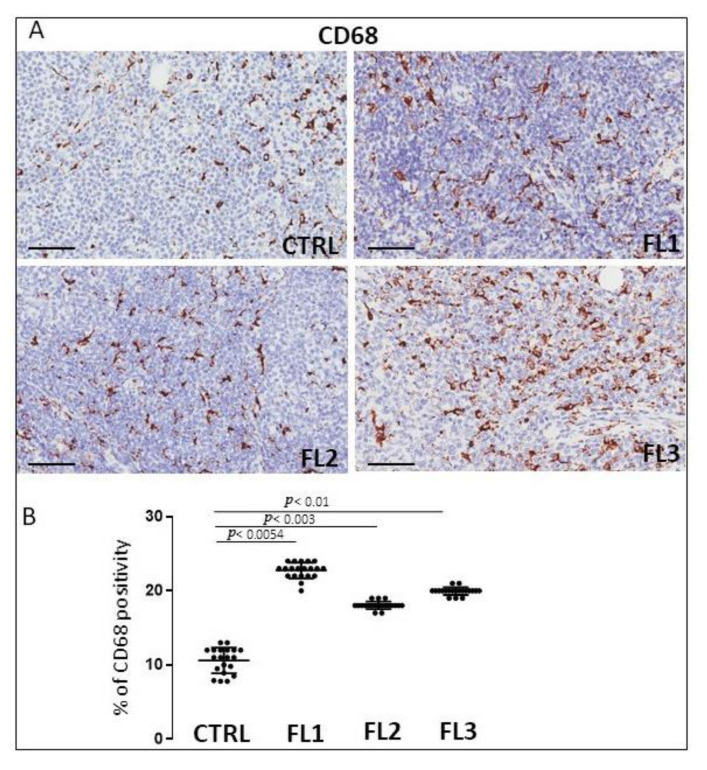
Immunohistochemical staining for CD68 in FL grade1 (FL1), FL grade2 (FL2), and FL grade 3 (FL3) and control samples (CTRL) (**A**). Scale bar: 60 μm (**A**). Morphometric analysis indicates the percentage of CD68 positivity in FL and CTRL samples (**B**).

**Figure 2:**
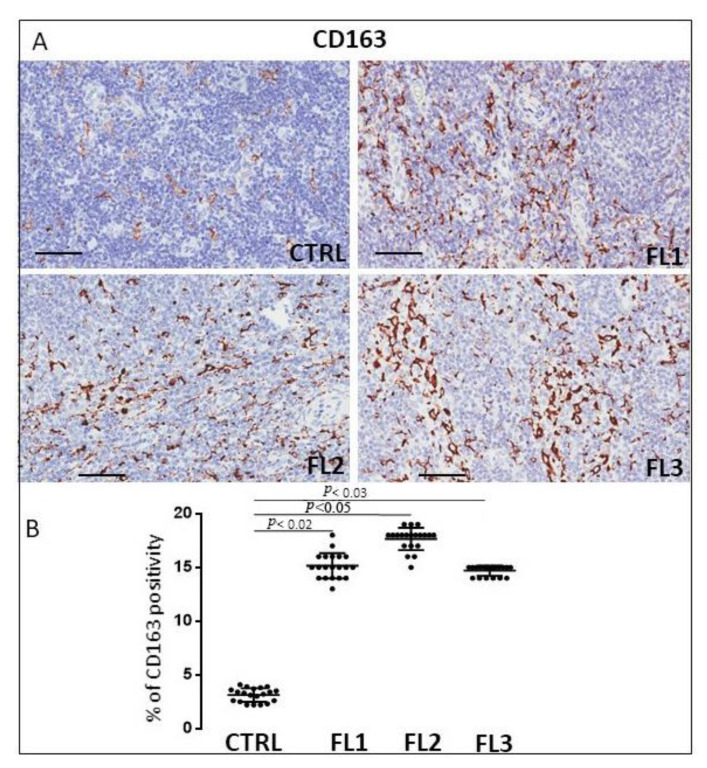
Immunohistochemical staining for CD163 in FL grade1 (FL1), FL grade2 (FL2), and FL grade 3 (FL3) and control samples (CTRL). Scale bar: 60 μm (**A**). Morphometric analysis indicates the percentage of CD163 positivity in FL and CTRL samples (**B**).

**Figure 3:**
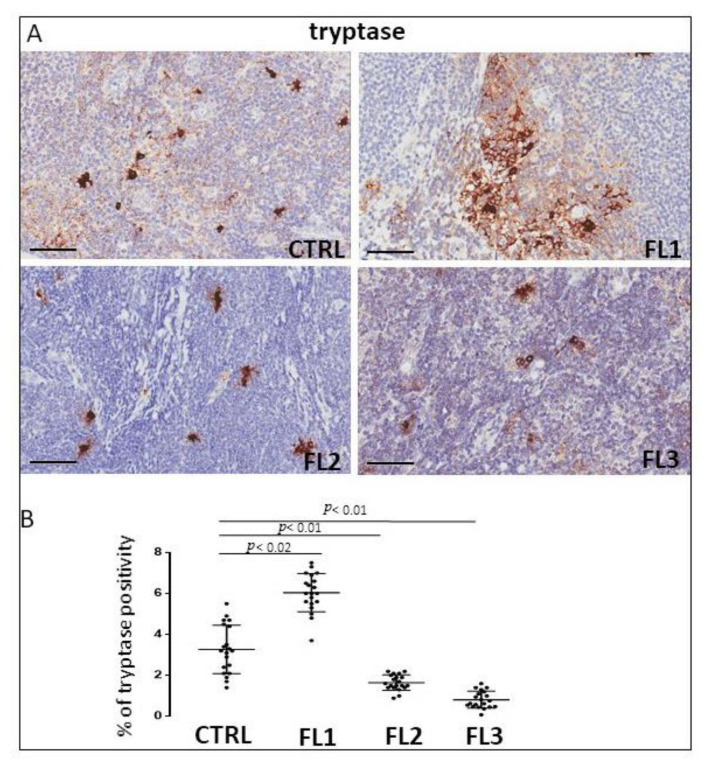
Immunohistochemical staining for tryptase in FL grade1 (FL1), FL grade2 (FL2), and FL grade 3 (FL3) and control samples (CTRL). Scale bar: 60 μm (**A**). Morphometric analysis indicates the percentage of tryptase^+^ mast cells in FL and CTRL samples (**B**).

Follicular lymphoma and CTRL tissue samples were immune stained for CD68 (Figure 1), CD163 (Figure 2) and tryptase (Figure 3) to evaluate total macrophages, M2 macrophages, and mast cells, respectively. Morphometric analysis showed significantly increased numbers of CD68^+^ and CD163^+^ cells in all the grades of FL samples (CD68: FL1 (22.4 ± 0.9%), FL2 (18 ± 0.4%), and FL3 (20 ± 0.46%); CD163: FL1 (14.8 ± 1.1%), FL2 (18 ± 1%), and FL3 (14.7 ± 0.4%) as compared to the CTRL group (CD68: 11 ± 1.6%; CD163: 2.9 ± 0.6%). Regarding tryptase^+^ mast cells, we observed their significant increase in the grade 1 of FL samples (tryptase: FL1 (6.1 ± 0.8%), FL2 (1.7 ± 0.4%), and FL3 (0.91 ± 0.41%)) as compared to the CTRL group (CTRL: 2.5 ± 1.4%).

### 3.2. CD4 and CD8 Immunohistochemistry

**Figure 4:**
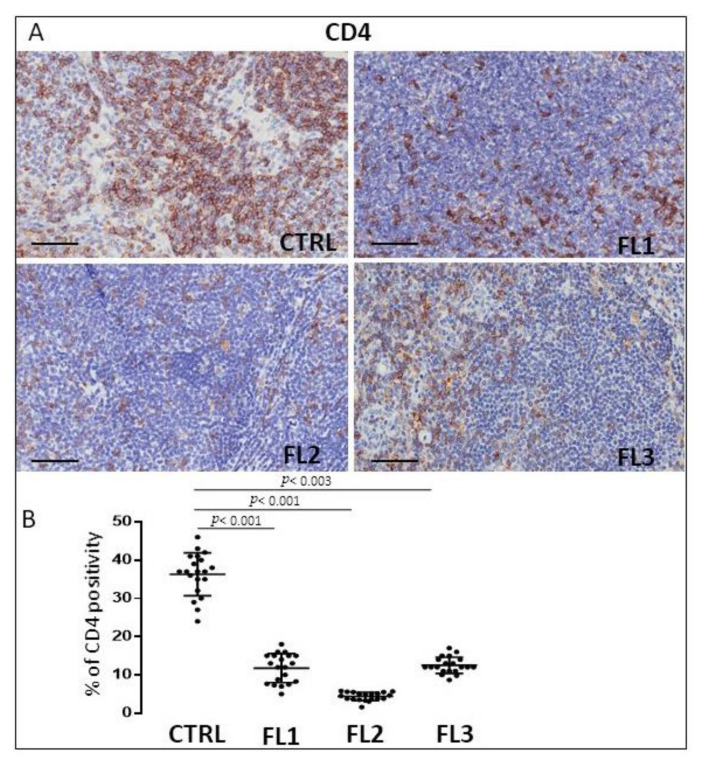
Immunohistochemical staining for CD4 in FL grade1 (FL1), FL grade2 (FL2), and FL grade 3 (FL3) and control samples (CTRL). Scale bar: 60 μm (**A**). Morphometric analysis indicates the percentage of CD4 positivity in FL and CTRL samples (**B**).

**Figure 5:**
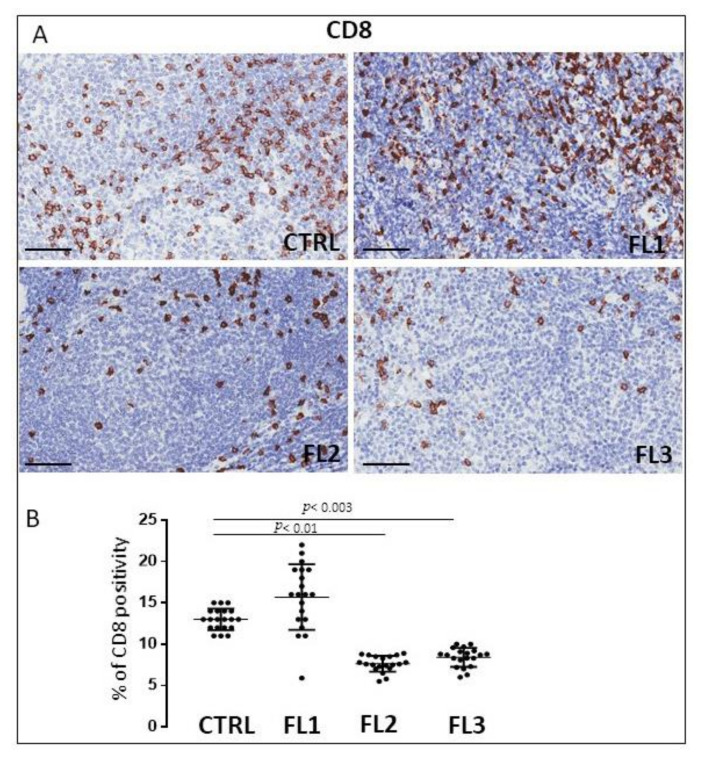
Immunohistochemical staining for CD8 in FL grade1 (FL1), FL grade2 (FL2), and FL grade 3 (FL3) and control samples (CTRL). Scale bar: 60 μm (**A**). Morphometric analysis indicates the percentage of CD8 positivity in FL and CTRL samples (**B**).

FL samples and CTRL specimens were immune stained for CD4 and CD8 to evaluate CD4^+^ (Figure 4) and CD8^+^ cells (Figure 5). Morphometric analysis indicated the significant reduction of CD4^+^ cell number in all the three FL grades (CD4^+^: FL1 (11.8 ± 3.2%), FL2 (4.6 ± 1.2%), and FL3 (13 ± 1.9%)) with respect to the CTRL (34 ± 6.3%). CD8^+^ cells number underwent a slight (not significant) increase in FL1 and a significant reduction in the other FL groups (CD8^+^: FL1 (15.3 ± 3.9%), FL2 (8 ± 1.1%), FL3 (8.4 ± 1.2%)) with respect to the CTRL (13 ± 1.3%).

### 3.3. CD34 and PD-L1 Immunohistochemistry

**Figure 6:**
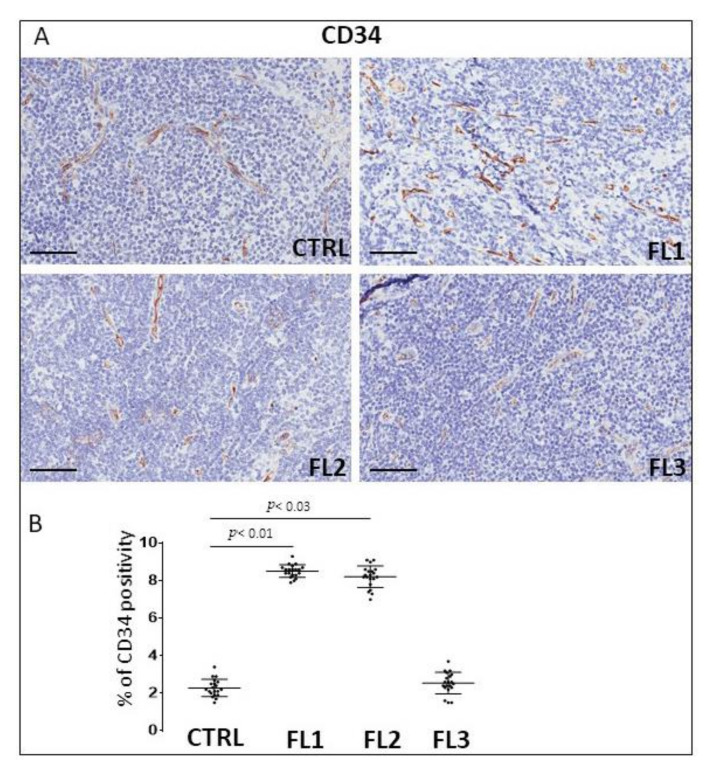
Immunohistochemical staining for CD34 in FL grade1 (FL1), FL grade2 (FL2), and FL grade 3 (FL3) and control samples (CTRL). Scale bar: 60 μm (**A**). Morphometric analysis indicates the percentage of CD34 positivity in FL and CTRL samples (**B**).

**Figure 7:**
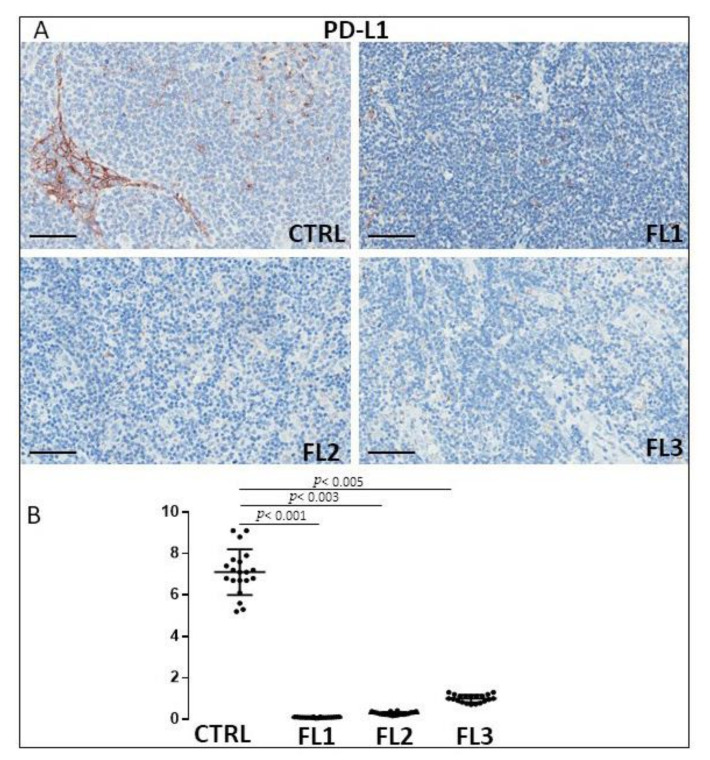
Immunohistochemical staining for PDL-1 in FL grade1 (FL1), FL grade2 (FL2), and FL grade 3 (FL3) and control samples (CTRL). Scale bar: 60 μm (**A**). Morphometric analysis indicates the percentage of PDL-1 positivity in FL and CTRL samples (**B**).

FL samples groups and CTRL specimens were immune stained for CD34 (Figure 6), and PDL-1 (Figure 7) to estimate the microvessel density and the number of PDL-1 expressing cells, respectively. Morphometric analysis showed the significant increase of CD34^+^ at grades 1 and 2 of FL samples (CD34: FL1 (8.6 ± 0.4%), FOLL2 (8.2 ± 0.54%); FL3 (2.63 ± 0.54%)) as compared to the CTRL group (CD34: 2.2 ± 0.5%). PDL-1^+^ cells, on the other hand, underwent a significant, strong reduction in FL groups (PD-L1^+^: FL1 (0.1 ± 0.02%), FL2 (0.3 ± 0.07%), FL3 (1 ± 0.2%)) respect to the healthy CTRL (7.3 ± 0.98%).

### 3.4. Correlation Analysis

**Figure 8:**
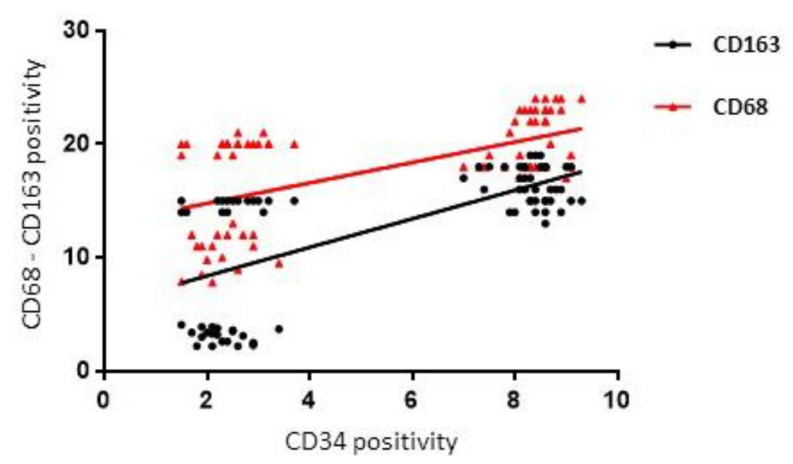
Regression analysis graph indicating the correlation between CD163 and CD34, CD68 and CD34 in FL and CTRL samples.

A positive correlation between CD68 and CD34 (rho = 0.52, *p* = 0.0001) and CD34 and CD163 (rho = 0.63, *p* = 0.0001) in the FL and CTRL groups (Figure 8) was found by Spearman correlation analysis.

No correlation was established between CD68, CD163, tryptase, CD4, CD8, CD34, PDL-1, and FLIPI (%) staging in FL patients

## 4. Discussion

In this work, we quantified by immunohistochemistry the inflammatory cell content and the microvessel density in tissue samples derived from the lymph nodes of FL patients at grades 1, 2, and 3A as compared with normal lymph nodes with the aim to establish a correlation with tumor progression. We selected patients with follicular growth patterns excluding the diffuse forms of FL in which the amount of interfollicular tissue is extremely variable and often absent, causing the back-to-back arrangement of follicles [44]. For this reason, we focused our analysis on the description of the characteristics of the follicular tumor tissue.

There is no effective therapy for FL, and after the considerable prognosis improvement with the introduction of the monoclonal anti-CD-20 antibody (rituximab) more than 20 years ago, FL prognosis has not undergone any substantial changes [45,46,47]. This is due to the marked heterogeneity of this tumor, which depends, in part, on the different characteristics of cellular infiltrate in the TME. The FL microenvironment is composed of tumor-infiltrating CD8^+^T cells, follicular regulatory and helper CD4^+^T cells, TAMs and mast cells, follicular dendritic cells, and reticular cells, all involved in the regulation of the prognosis and progression of FL.

TAMs are involved in tumor growth, angiogenesis, metastasis, and immunosuppression in different types of lymphomas [48]. In particular, the association between TAMs and survival in FL progression is still controversial [49,50,51,52]. Kridel et al. reported that the increased CD163^+^ cells were predictive of adverse or favorable FL outcomes dependent on R-CVP, or R-CHOP/R-maintenance treatment received [52]. Additionally, Dave et al. demonstrated that the downregulation of genes related to macrophage function predicts a favorable outcome [51]. Canioni and collaborators observed that patients with a high number of TAMs presented a significantly reduced event-free survival [50]. Some authors considered CD68 immunostaining as a useful routine tool in the prediction of the outcome of FL patients with a high tumor burden who receive chemotherapy [53]. In this study, we demonstrated an increased number of CD68^+^ and CD163^+^ TAMs in FL grade 1 which remains constant in 2 and 3A of FL samples with respect to the normal lymph nodes.

Cancer progression is strongly influenced by the crosstalk between the tumor cells and inflammatory cells and both are associated with angiogenesis [54]. An increased number of blood vessels indicate a poor prognosis and low rate of survival in FL and clinic-pathological data indicate angiogenesis as a target for therapy [55]. In FL patients with heterogeneous treatments, the increased interfollicular microvascular density predicted inferior overall survival (OS) and increased transformation to DLBCL [56]. These findings suggest that microvascular density may provide a rationale for trials with anti-angiogenic molecules in FL patients with increased microvascular density [55,57]. Our observations indicate an increase of microvascular density in grades 1 and 2 of FL samples as compared to the controls. The positive correlation between the increase of TAMs and microvascular density confirms the pro-angiogenic role played by TAMs in the tumor microenvironment of lymphomas [58,59]. Mast cells have a crucial role in tumor angiogenesis [18,55,60], and their number is increased in bone marrow [61] and lymph node biopsies of NHL patients [62]. High mast cell counts interfere with the outcome of immune-chemo-therapy-treated FL patients [49], and the adverse prognostic significance of MCs was demonstrated both in the treated patients and patients at diagnosis [63]. Our findings indicate that the tryptase^+^ mast cell number resulted in an increase only in the grade1 of FL samples, whereas in grades 2 and 3A, their number decreased with respect to the controls. We did not find a correlation between the microvascular density and mast cell numbers in FL. A high number of CD8^+^ T cells has been correlated to good prognosis [64,65] and a high number of CD4^+^ T cells to poor prognosis in FL [66,67], even if the prognostic importance of T cells infiltration seems to be modified by the therapy [65,68]. Our data indicated a reduction of CD4^+^ cells in all three FL grades with respect to the controls. Concerning CD8^+^ cells, their numbers resulted in an increase in grades 2 and 3A of FL with respect to the controls, whereas in grade 1, the CD8^+^ number did not significantly increase with respect to the controls. The decrease of the CD4/CD8 ratio could contribute to tumor angiogenesis and immune evasion during the late FL stages, as we have previously demonstrated in DLBCL and in MZL, where a positive correlation between the decreased CD4/CD8 ratio and microvessel density was established [16,18]. PD-1 [69] signaling induces the inhibition of T cells after ligation with PD-1 ligands (PD-Ls) such as PD-L1 [70,71] or PD-L2 [72], on neoplastic cells and in the tumor microenvironment. PD-L1 expression has been associated with clinical response to a PD-1 blockade in many clinical trials. Most FL tumor cells do not express PD-L1 or PD-L2 [73,74], however, PD-1^+^ cells are abundant in the TME of FL [73] and include T cells and follicular helper T cells from lymphomatous follicles or residual germinal centers [75]. In our study, we observed a significantly reduced presence of PD-L1^+^ cells in all the grades of analyzed FL samples.

## 5. Concluding Remarks

Our study has shown an increased number of CD68^+^ and CD163^+^ TAMs in FL grade 1 which remains constant in 2 and 3A of FL samples with respect to the controls and an increase of microvascular density in grades 1 and 2 of FL samples as compared to the controls. The positive correlation between the increase of TAMs and microvascular density confirms the pro-angiogenic role played by TAMs in the tumor microenvironment of lymphomas. Concerning mast cells, our findings indicate that the tryptase^+^ mast cell number resulted in an increase only in grade 1 of FL samples, whereas in grades 2 and 3A, their number decreased with respect to the controls; we did not find a correlation between microvascular density and mast cell number. Concerning T cells, our data indicated a reduction of CD4^+^ cells in all the three FL grades with respect to the controls, whereas the number of CD8^+^ cells resulted in an increase in grades 2 and 3A of FL with respect to the controls; additionally, the decrease of the CD4/CD8 ratio could contribute to tumor angiogenesis and immune evasion during the late FL stages. Finally, we observed a significantly reduced number of PD-L1^+^ cells in all the grades of analyzed FL samples, establishing an inverse relationship between the number of PD-L1^+^ cells and tumor progression in FL.

Overall, these data confirm that the increase in TAM infiltration in FL lymph nodes is correlated with the increased microvascular density, underlying the crucial role played by these cells in lymphoma TME. Over the last 30 years, the importance of angiogenesis in human lymphoma has become well recognized, and several factors involved in its control are being identified. Anti-angiogenic therapy is an important tool for the treatment of human lymphoma. However, a significant number of patients are resistant, whereas those who respond have minimal benefits. Further research should provide new useful therapeutic approaches and increase options for patients with resistant or refractory disease. Finally, correlation analyses on a very large case series could provide results that would allow the development of new prognostic indexes significant for FL stratification.

## Figures and Tables

**Table 1:** Main clinical features of FL patient.

FL Patients		
Gender (%)	Male Female	60 40
Age (years)	Median Range	62 39–80
Ki67%	Ave Range	27 10–70
Stage (number)	I II IIIA	20 20 20
First diagnosis (%)	I II IIIA	100 100 100
Extranodal sites (%)	I II IIIA	0 0 0
FLIPI (%)	1 2 3	26 60 14
